# The Function of Memes in Political Discourse

**DOI:** 10.1007/s11245-024-10112-0

**Published:** 2024-11-06

**Authors:** Glenn Anderau, Daniel Barbarrusa

**Affiliations:** 1 University of Zurich, Zurich, Switzerland; 2 University of Seville, Seville, Spain

**Keywords:** Social epistemology, Epistemology of the internet, Memes, Epistemic injustice, Extremism, Misinformation

## Abstract

The use of memes has become increasingly widespread in political discourse. However, there is a dearth of philosophical discussion on memes and their impact on political discourse. This paper addresses this gap in the literature and bridges the divide between the empirical and philosophical work on memes by offering a functionalist account which allows for a more in-depth analysis of the role memes play in political discourse. We offer a taxonomy of the eight key characteristics of memes: 1. humor; 2. fostering in-group identity; 3. caricatures; 4. replicability; 5. context collapse; 6. hermeneutical resources; 7. low reputational cost; 8. signaling. On the positive side, the propensity memes have to foster in-group identity and to function as a hermeneutical tool for people to make sense of their own experiences are a boon especially to marginalized communities. On the flipside, the creation of an in-group/out-group dynamic can also be exploited by sinister political actors, especially since the low reputational cost of circulating memes allows for plausible deniability. We use the analysis in this paper to jumpstart a discussion of how we should understand memes and debate which norms should govern the novel speech act of posting a meme given its impact on political discourse. Based on our findings, we end with a call to adopt stricter norms for the act of posting a meme.

## Introduction

Memes play an increasingly large role in our online communication. Anyone who is a frequent user of social media is likely to encounter memes on a regular basis and may even post memes themselves. And memes are not just restricted to purely innocuous debates: Memes are being used more frequently in political discussions online as well (Al-Rawi 2021; Denisova 2019; Lynch 2022). Despite this, there is a remarkable dearth of philosophical literature on the topic. Most importantly, there is even less work in philosophy which explains why memes have managed to play such a large part in political discourse. This paper aims to rectify this issue by proposing a functionalist account of memes. We use this account to show the features distinctive of memes, while not exclusive to political memes, can make them a valuable tool to convey political messaging. We then explore both the positive and the negative effects memes can have on political discourse. In a final step, we consider what should be done to amplify the positive effects of memes while curtailing the negative ones. This involves rethinking the norms which govern new speech acts such as posting a meme. Given the massive impact of memes in our everyday lives, including political debates, we believe that the norms of meme usage need to reflect their outsized impact. Another goal of the paper is to bridge the gap between the philosophical literature and the much more extensive work on memes in other academic disciplines.

## Existing Literature

The term *meme* was coined by Richard Dawkins in his 1976 book *The Selfish Gene* (Dawkins 1976). Dawkins’ conception of the term meme predates the internet meme and is not exactly synonymous with it, although the two conceptions of memes do dovetail nicely. For Dawkins (1976), memes are *units of cultural transmission* or *units of imitation/replication*. What genes are to biology, memes are to culture according to his view. In this sense, memes can be anything from “tunes, ideas, catch-phrases, clothes fashions, ways of making pots or of building arches” (Dawkins 1976, p. 206). This original, broader notion of meme saw important developments thanks to Susan Blackmore (2000). The first person to co-opt the term ‘meme’ in order to describe internet memes was Mike Godwin (1994).

While the etymology of the word ‘meme’ is derived from Dawkins, there are some important differences between memes as described by Dawkins and internet memes.^1^ While memes in Dawkins’ understanding are prone to mutation, these changes happen unintentionally and much more slowly than in internet memes. A meme in this sense is still a longer lasting unit of cultural transmission. Mutation is something which happens deliberately in internet memes, which are usually much shorter lived than the memes Dawkins had in mind. Meme templates for internet memes are meant to allow for a myriad of different versions of the same meme, which means that changes in their content are not unintended errors, but purposefully sought out.

Although some features of Dawkins’ memes are transferable to the narrower internet memes (i.e. replicability), paradigmatic features of the latter, like their humoristic nature, are totally absent in the classical notion. In this paper, we are exclusively interested in internet memes. From here on out, all mentions of the word ‘meme’ should be understood as ‘internet meme’ (unless otherwise specified).

Before developing our account, we need to take stock of the existing philosophical literature on memes. When it comes to philosophy, there is some discussion of Dawkins’ (1976) classical notion of *meme*.^2^ When it comes to internet memes, there is a large body of research in communication, mainly under the prism of digital culture studies, that has not fully transferred to philosophy yet.^3^ This is unfortunate: except for Scott (2021) and Lynch (2022), there is a notable absence of philosophical work on internet memes. Thus, while communication scholars analyze, for example, how memes leverage background knowledge to produce humor (Diedrichsen 2022), or how they push political agendas (Schmid et al. 2024), it is not their goal to address philosophical questions on the normativity of sharing memes as a speech act, or on how knowledge is produced or eroded through these activities. We aim to bridge this gap and bring the discussion of *internet memes* to philosophical ground.

The philosophical literature on internet memes is still in its infancy. There are two accounts which we wish to highlight here: One by Kate Scott (2021), who aims to analyze memes as a novel speech act and another by Michael Lynch (2022), who tries to see how sincerity conditions can apply to memes which contain political messaging. Scott (2021) proposes that memes are *multimodal metaphors*. While they combine different media (e.g. text and image), they function the same as metaphors and are understood the same way we understand verbal metaphors (Scott 2021). Therefore, we do not need a unique framework to explain how we understand memes according to Scott (2021).

**Fig. 1:**
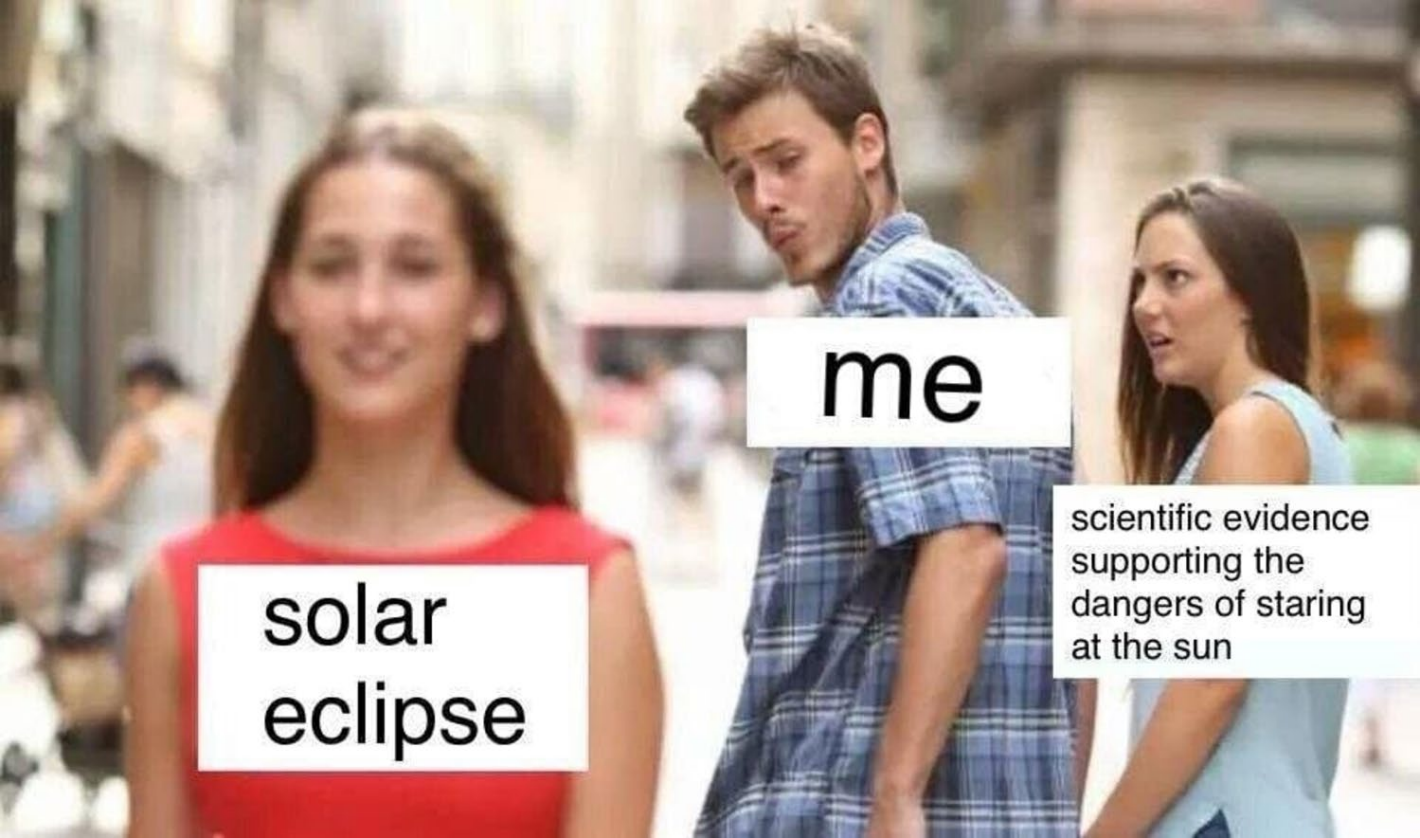
‘Solar eclipse’ labels over ‘Distracted Boyfriend’ template. Original photograph by Antonio Guillem/Shutterstock.com

Her analysis focuses on a specific type of meme, the object labeling meme (see Fig. 1, an example discussed in her paper). In these types of memes, we have an image which is adorned by text boxes labeling items in the image (in the case of the distracted boyfriend meme, this would be the distracted boyfriend, his partner, and the woman in red). While object labeling memes are a very common form of meme, it should be noted that there are memes utilizing different media, such as gifs or videos for example. It is plausible that Scott’s (2021) analysis could extend to many of these memes as well, but this is not a claim she is committed to in her paper.

Lynch (2022) is one of the only voices in the philosophical literature who tries to analyze the impact memes have on political discourse, highlighting the potential use of memes in disseminating misinformation. Addressing this issue is tricky according to Lynch (2022), since it is unclear how sincerity conditions could apply to memes in the first place. Lynch (2022) solves this by claiming that memes do have a clearly understandable (political) meaning they are trying to convey and by clarifying that even though we cannot know if posters *believe* in this content epistemically, they can still *commit* to it politically. Political commitments in this sense are some kind of non-epistemic endorsement, in these cases of a meme (Lynch 2022). While it is impossible to determine whether people really believe in the message of the memes they post, we can still apply sincerity conditions to memes in terms of their political commitment (Lynch 2022).

## Motivation & Focus on Political Memes

Given the relatively sparse philosophical literature on memes, we felt it was particularly important to make sure our own work can lay the groundwork for the future discussion on memes and their political impact. As such, our goal is not to quibble over the minute details of the correct definition of memes with Scott (2021) or pick apart Lynch’s (2022) proposal on how sincerity conditions can apply to memes. Rather, we would like to take these two works as a basis for our own analysis while trying to expand upon some of the questions which cannot be fully answered by the existing literature. Most importantly, we want to defend the view that memes are extremely powerful tools for political messaging and examine how this power can be used both fruitfully and exploited by more nefarious actors. To do so, we will put forth a functionalist account highlighting 8 key characteristics essential to memes. While these functions apply to memes in general, we also believe that all of these functions help memes play an important role in political discourse. Memes are not necessarily political, but their key characteristics are all useful for conveying political or ideological messaging. Being political itself is not a necessary condition of memes of course: Memes can still be apolitical and many of them are. Before we get to our account, we want to offer a few clarifications:

### Why Focus on Memes in the Context of Political Discourse?

Our main motivation for analyzing the use of memes in political discourse is that we believe that this is where memes are having the biggest impact. If there are normative questions which arise from new speech acts such as posting memes, then they are most likely to concern how memes are used as vehicles for political messaging. We share Lynch’s (2022) concern about memes being misused in this regard. At the same time, we also want to account for positive contributions memes can make in the political realm, a factor which we believe should not be dismissed too lightly and is not explored in the literature at all. Nevertheless, we believe that our analysis of memes can contribute to the much larger philosophical literature discussing misinformation and how to stymie its spread. Given this motivation, it makes sense to focus on memes in the context of political discourse. The focus on political memes has a practical advantage as well: Since memes are a large and heterogeneous phenomenon, narrowing our sights to political memes helps us sharpen our analysis. However, it should be noted that the functions described in our account apply to all memes, not merely political memes.

While our analysis will focus on political memes, we do not want to limit the scope of the paper too far. This means that we are operating with a broad understanding of the term ‘political’. This includes memes which are not *explicitly* political or do not involve political institutions or figures directly. They need not concern themselves with party politics. It is enough that memes can in any way be interpreted as political or ideological. If in doubt, our heuristic would be to err on the side of including a meme on the side of the political. Based on our very lax understanding of *political* it would not even be necessary to speak of ‘sides’: We treat the distinction between ‘political’ and ‘non-political’ as a gradual rather than a categorical one. We agree with Lynch’s understanding (2022) of what a political meme is, as well as his discussion of the political meaning and commitment of memes.^4^ Importantly, the functions of memes we describe apply to memes tout court, which means that the distinction between political and non-political memes is not relevant to our account itself.

### Why Not Offer a More Clear-Cut Definition of ‘meme’?

One open question is why we opt for a functionalist account rather than a more strict definition of the term *meme* which lists the necessary conditions which need to be fulfilled before we use the term. Given the ever-changing nature of memes, we actually view this as an advantage of our view. Memes not only allow for but actively seek out changes in their form and content. As such, memes are a constantly evolving speech act and their form (including which kinds of media we are willing to consider as constituting memes) are prone to change. The danger of a classical philosophical definition is that it would be too rigid to account for any potential evolutions of what is commonly understood as a ‘meme’. The characteristics we try to highlight in our account are designed to remain relevant even if some of the formal aspects of memes will change in the future. Our functionalist account is less rigid and malleable enough to adapt to future variations of memes, which are likely to develop in the future.^5^

One potential worry could be that our account becomes harder to operationalize without giving strict formal criteria. We can see how it could be extremely useful to provide more stringent formal requirements for practical reasons in domains such as politics or law if one is interested in regulating the use of memes from a legal perspective: A law on memes could simply focus on the most dominant or permanent type of meme and this could be defensible from a practical standpoint. Unfortunately, the downside is that any definition with such strict conditions is likely to be (a) limited (because it only deals with a specific type of meme, as is the case in Scott 2021), (b) imprecise (because it excludes niche forms of memes), (c) prone to becoming outdated in the future (because the most popular form of memes changes).

We do believe that these downsides can be outweighed by practical reasons depending on the purpose and context of another account of memes. However, given the fact that this is still a theoretical and philosophical paper, these costs seem too steep for our own account of memes. If anyone wishes to operationalize our account, they can do so by either adding strict formal criteria for memes (for example, by focusing solely on object labeling memes) while still using the functions of memes we describe.

**Fig. 2:**
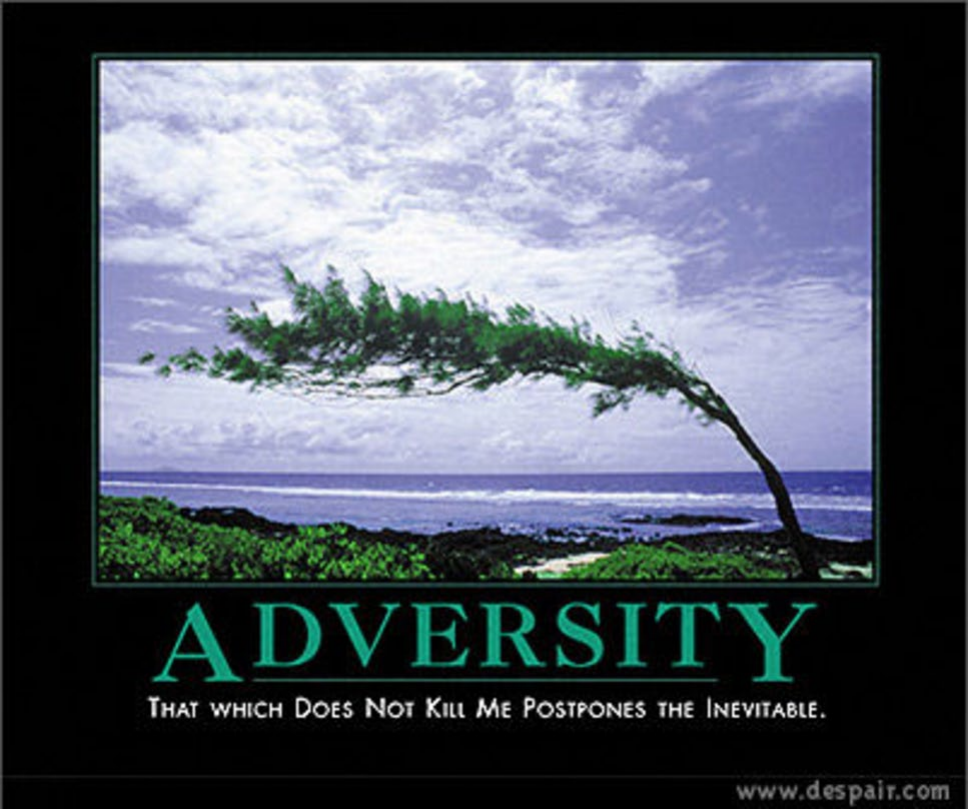
Example of ‘Demotivator’. Found in W K (2009)

**Fig. 3:**
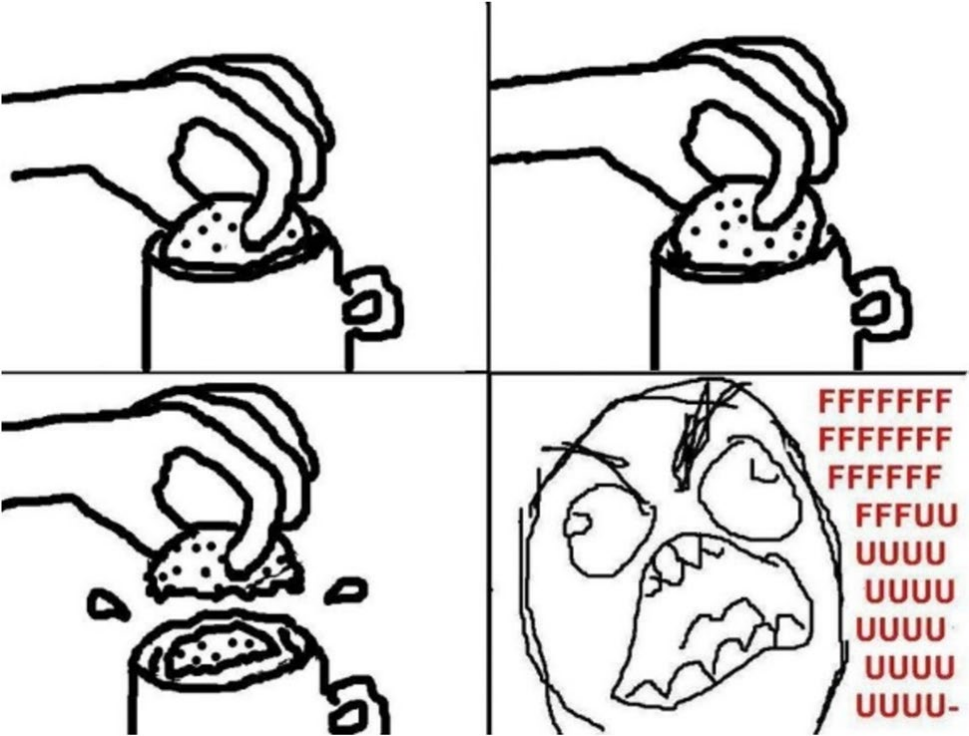
A rage comic

**Fig. 4:**
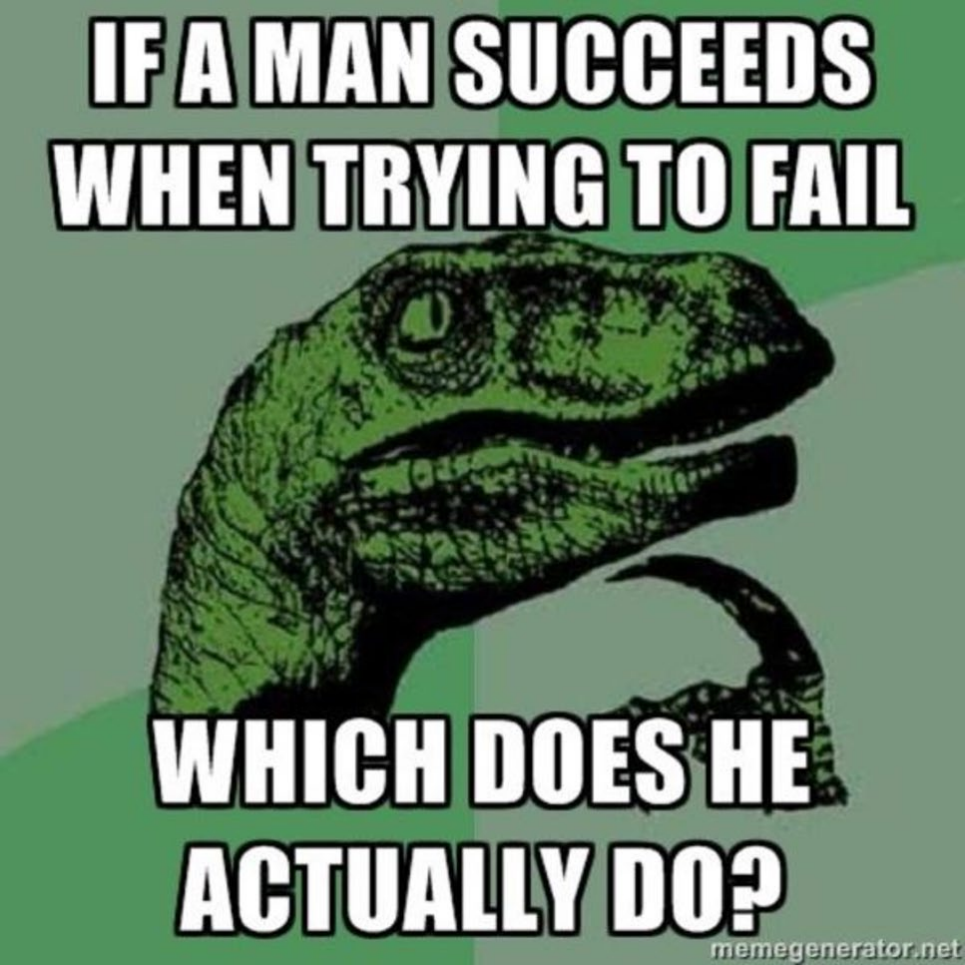
‘Philosoraptor’

Consider a brief history of how fast the paradigmatic cases of memes have changed. In the early 2000s, one of the most popular use of memes was that of “demotivational” images (W K 2009, see Fig. 2). This trend would be succeeded by that of “rage comics”, which would combine poorly drawn vignettes, along with some text, and one or several “rage faces” (see Fig. 3 above). In the same time period, ‘Advice animals’ were also immensely popular—these associated a certain background template, a character, and one type of joke. ‘Philosoraptor’, for example, was supposed to make amusing and witty questions (Fig. 4). These trends are long obsolete and largely forgotten. If we had insisted back then on specific criteria to count as a meme (e.g.: “a meme needs to present a template that resembles a motivational picture”, or “a meme needs to depict a poorly drawn vignette”), we would miss out on much of this evolution. While object-labeling memes (like Fig. 1 above) and when-memes are immensely popular now, it remains to be seen how long they can retain this status before other meme formats become more popular. In fact, videos have become a much more prominent medium for memes, as is exemplified by the popularity (especially with younger audiences) of the ‘Skibidi Toilet’ web series (DaFuq!?Boom! 2023, Fig. 5). Because our goal is to develop an account which is resistant to changing trends, we believe it is more accurate to focus on the functions memes have regardless of the format they use. We believe that such a definition is more valuable in the long run, since it can hold even as popular meme formats change.

**Fig. 5:**
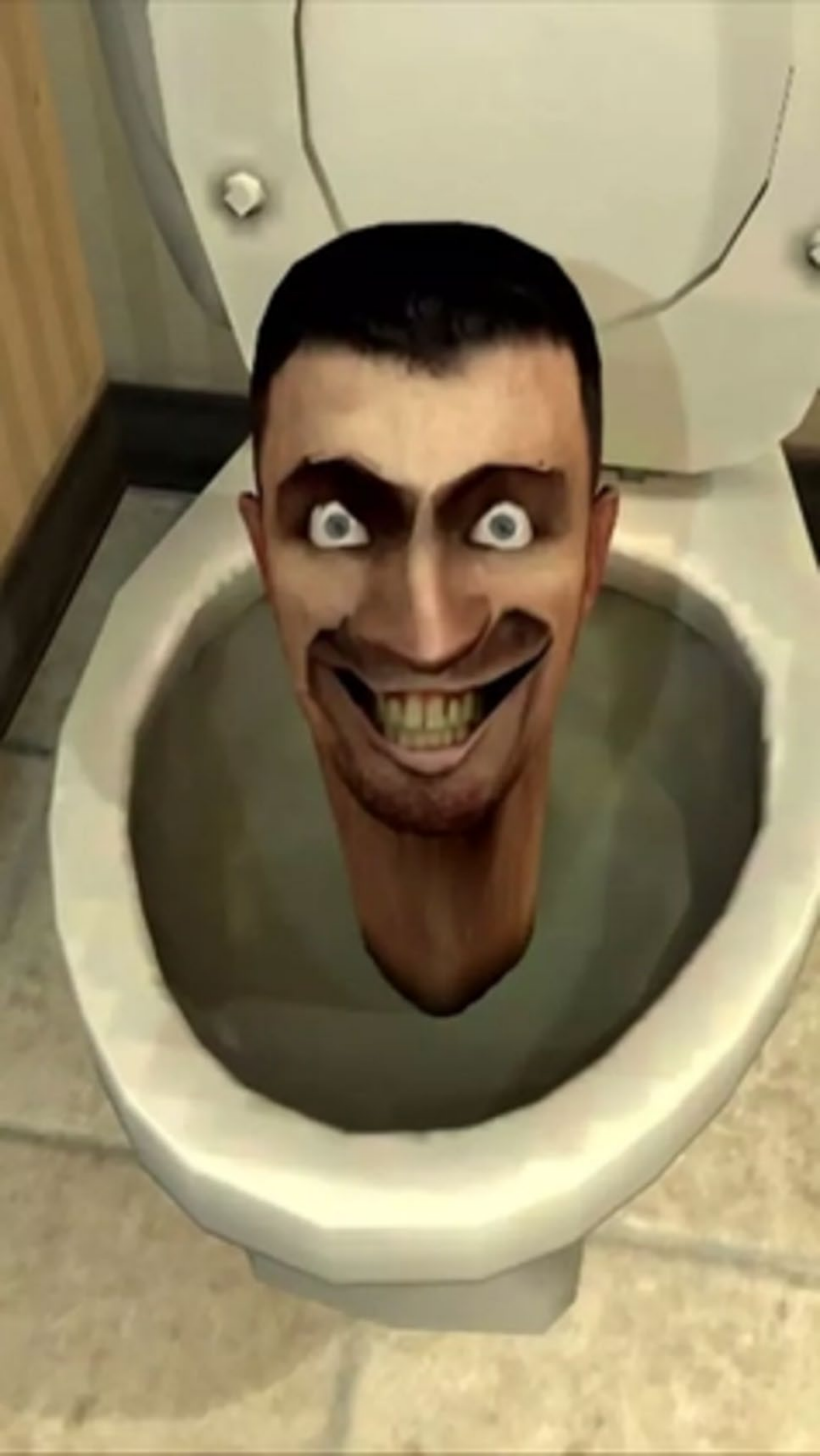
Skibidi Toilet (still image from video)

## The Functionalist Account of Memes in Political Discourse

As already mentioned, we want to develop a functionalist account of memes. The approach here is to outline the paradigmatic functions of memes and show how all of them could be useful within political discourse (even though the functions apply to memes even if they are not political). This approach is very much inspired by the work of Don Fallis on *disinformation* (2015).

Two points are especially relevant from Fallis' work. First, he defines disinformation as misleading information that has the function of being misleading (Fallis 2015, p. 413). He goes on to define a function as “the action for which a person or thing is particularly fitted or employed” (following American Heritage 2000).^6^ Since a function is embedded in the phenomenon itself, the notion helps to detach the studied phenomenon from the user’s intentions, while at the same time recognizing that its effects are not random or casual. For instance, in the case of disinformation, Fallis rejects that disinformation is produced by sharing information *with the intention* to mislead: that would be too narrow, because one can clearly share disinformation even when one has no intention to mislead. In the case of memes, we’ll see how certain functions, like fostering in-group identity or signaling certain political commitments can work even if the user just had the intention to engage in humor.

Second, functionality is historical. A function can be acquired either by design or by evolution (Fallis 2015, following [Graham 2010, pp. 153–155; Krohs and Kroes 2009]). Again, the role of intentions is somewhat transformed in this framework. A meme, for example, can have the function to signal belonging to a political group by design (e.g.: it contains clearly identifiable symbols) or by evolution (even if the meme was originally non-political, the way it’s been shared and consumed through time has transformed it into a partisan symbol, e.g. Pepe the Frog).

It should also be noted that not all of the characteristics we highlight are functions themselves: Some of them are merely attributes memes possess or background conditions which help memes play a role within political discourse.^7^

We use Scott’s (2021) aforementioned account as a starting point. This means that memes are multimodal^8^ metaphors: They (often) combine different media and are read by us as metaphors or analogies. In addition to this, we highlight eight key features of memes which help them fulfill a role in political discourse. One important question which needs to be clarified in advance is whether a meme needs to be widely spread and disseminated in order to be considered a meme or whether an individual meme which has not gone viral or been shared a lot would qualify as a meme too. On our account, it is not necessary for memes to ‘go viral’ and become widely shared in order to be considered memes as such a requirement would be unnecessarily restrictive. It would also require us to quantify a threshold of virality or spread which needs to be reached in order to qualify as a meme and any such limit would be arbitrary. Nevertheless, there is one caveat to this: We do require memes to at least have the inherent potential to be widely spread and disseminated in order to be considered a meme. At the moment, this potential is fulfilled if memes are posted on social media or at least online, since these platforms allow for the possibility of widespread dissemination and replication of a meme template. While this means that we see memes as a largely online phenomenon at the moment, we do not want to rule out that there could be platforms outside of the internet which have the inherent potential for large scale diffusion as well. This is especially important if we want to account for future variations of memes, which could plausibly be spread on different platforms. For the time being, however, it is safe to think of memes as a form of communication which is heavily tied to social media and the internet.

In total, we outline 8 key functions of memes. These can be separated into three categories (see Table 1): (1) Humor, (2) Conditions of production/spread, (3) Conditions of reception. We will go through all these features one by one. While we believe that these functions apply to memes in general, we will also outline how each function makes memes a useful tool for political discourse.

**Table 1:** The eight functions of memes

*Humor-related functions*	
1. Humor	
2. Fostering In-Group Identity	
3. Caricatures	
*Conditions of production-spread*	
4. Replicability	
5. Context collapse	
*Conditions of reception*	
6. Hermeneutical resources	
7. Low reputational cost	
8. Signaling

1. Humor
  1. Humor

One of the most common features noted about memes is that they are often, if not always, humorous in nature (Lynch 2022; Mortensen and Neumayer 2021). While memes might be read as metaphors or analogies, they are also tongue-in-cheek and meant to be funny as well. It makes sense to compare memes to forms of humor, such as jokes. Similarly to jokes, memes seem to rely on the audience sharing some of the same background information, beliefs or experiences in order for them to be understood properly. ‘Getting’ a joke is similar to ‘getting’ a meme: It relies on shared knowledge of the topic matter and by explaining the context post-hoc, we ruin the joke or the meme.

Ted Cohen (1999) puts forth an account of jokes which claims that they rely on assumed shared background knowledge. Telling a joke according to his view is always a leap of faith: The joke can only be successfully understood by the audience if they share the same background knowledge (necessary to understand the joke) with the joke teller. But this shared knowledge cannot be preemptively ascertained, since doing so would telegraph or ruin the joke. While there is always a risk for the joke teller, this leap of faith is rewarded when the audience does share the background knowledge necessary to understand the joke: According to Cohen (1999), in the cases where the risk pays off, jokes create a sense of *intimacy* between the audience and the joke teller. Thi Nguyen (2021) co-opts this framework to argue that Twitter is both an amazing platform to create intimacy in Cohen’s sense while at the same time undermining said intimacy. This is because the platform’s design makes it so prone to context collapse, which makes it likely that the jokes told on Twitter will be seen outside of the intended audience which would be most likely to possess the shared background knowledge.

We believe that Cohen’s (1999) and Nguyen’s (2021) reading of jokes perfectly maps onto memes as well. Memes also rely on shared assumed background knowledge in order to be understood. They can voice sentiments which the audience agrees with but may not have been able to express themselves. The intimacy which Cohen (1999) ascribes to jokes can be transmitted by memes as well. The background knowledge needs to be instilled in the audience prior to being exposed to the meme: Explaining it afterwards ruins the humor contained within a meme the same way in which explaining a joke would. In this sense, we can co-opt Cohen’s (1999) reading of jokes to account for the humorous nature of memes as well.

One might wonder if we should just conclude that memes are a type of joke themselves. While we do not completely rule out this option, we think there are many benefits to thinking about memes as a category of their own. For one, there are many formal characteristics which apply to memes which do not apply to jokes at all, such as their multimodality or the fact that they employ the structure of metaphors and analogies. Jokes are to be told; memes are to be shown. We believe that the visceral nature with which memes can make a point through their combination of different media is particularly important. A similar thing is true for the conditions of their spread, which mainly takes place online,^9^ which is too restrictive for the category of jokes. As such, we think it is best to treat memes and jokes as two categories which share a strongly overlapping Venn diagram, but which are both worthy of being studied in their own right. However, since this paper is not directly concerned with the theory of jokes or humor, we would be open to an account which treats memes as a very specific type of joke, as long as the many differences are properly accounted for.

2. In-group identity

One of the main benefits of memes as a tool for political discourse is that they are extremely effective at fostering an in-group identity among their (target) audience. We believe that this feature is heavily reliant on the sense of intimacy described in the section above which memes can create through their humorous nature. If the audience shares the same background knowledge or the necessary experiences to understand and relate to the meme, it can automatically make the audience feel a sense of inclusion. This seamlessly creates an in-group/out-group dynamic in the audience of a meme. Fostering a strong sense of in-group identity (and conversely, separating oneself from the out-group) are extremely valuable effects for anyone engaged in political discourse. This makes memes a valuable tool for political discussions.

Highlighting shared background knowledge or experiences through the form of memes is a more effective tool than simply trying to ascertain whether it exists through more traditional modes of communication, such as asserting or explaining it to the audience. Memes can strengthen in-group identity through ‘showing, not telling’, to borrow an analogy from literary theory. As such, memes become valuable for political discourse not only because they can strengthen an in-group/out-group identity, but because they are more effective at doing so than other forms of communication. Using memes becomes a ‘high-reward’ communicative strategy within political discourse.

3. Caricature

A third point relating memes to humor is that they can function as caricatures of (political) viewpoints, both one’s own but often also of opposing views. While memes lend themselves to making political points, their messaging will necessarily need to be condensed to fit within the meme format. Although some meme templates allow for relatively large textboxes, they hardly allow for lengthy debate or elaboration. For memes to effectively communicate a political point, they need to ensure that this point is one which can easily be explained and grasped within the confines of a meme. As such, memes depicting political viewpoints will struggle to add a lot of nuance to the points they are making. Arguably, the introduction of too much nuance would curtail their ability to make political points effectively and dilute their messaging.

The caricature function of memes and the removal of nuance which comes with it need not necessarily be seen as a negative thing. Caricatures can exist in political discourse outside of memes and be a great tool for making concise points in the most effective manner possible. For instance, we can think of the role political cartoons in newspapers play within political discourse: They add witty social commentary on topics without providing lengthy explanatory background information. Memes could be seen as fulfilling a similar role when they consist of caricatures of political viewpoints. They can be a tool for legitimate political satire.

But there is a flipside to memes acting as caricatures as well. One cartoon serving as political satire in a newspaper is fine, but we would not want newspapers to largely consist of caricatures either. With memes playing an increasingly large role within political discourse online (Lynch 2022), the ratio of caricature to more sober, informative discussion of political topics is threatening to shift. In this sense, memes play a very worrying role in removing nuance from complex political debates, not necessarily on an individual basis, but as a whole if they make up too large of a percentage of our (online) political discourse.

And there is another pernicious effect memes can have if they devolve into unflattering caricatures of opposing political viewpoints, namely that they can degrade political discourse and drive polarization. Imagine you encounter a meme which presents a caricature of a political viewpoint which you hold. The version of your view depicted in this meme is a mere strawman, used only to be knocked down and mocked by the meme. What is the correct way to respond to such a meme, especially if confronted with not only an individual instance of it but a barrage of similar memes? This question is of course rhetorical. There does not seem to be a particularly constructive manner in which one can respond to such discourse. There is no avenue which allows for a good-faith engagement with memes which depict caricatures in this way. The only avenue to respond would be in kind, with a meme of one’s own offering a caricature of the opposing viewpoint. This leads to even more polarization. Many political debates online have descended into ‘meme wars’ (Al-Rawi 2021; Bogerts and Fielitz 2018; Denisova 2019; Hannan 2018; Lynch 2022), which shows that the worries concerning the negative impact of the caricature function of memes are not merely hypothetical: Memes are already contributing to the breakdown of political discourse and polarization now. This effect can further contribute to the in-group/out-group dynamic discussed in the section above as well.

The existence of ‘meme wars’ also highlights the next characteristic we will discuss below: *replicability*. Meme wars can easily include a vast number of participants because of the conditions of their spread online and the ease with which memes and meme templates can be replicated. This highlights how memes are different from conventional caricatures: It seems strange to consider the existence of a ‘caricature war’ and if there was such a thing, it would take place between a relatively small number of artists who are in the relatively privileged position to reach an audience with their caricatures. Meme wars, however, can have an almost limitless number of participants online, since the hurdle for posting a meme on social media is extremely low. We discuss the replicability of memes and the conditions of their spread in the next section below.

2. Conditions of production/spread
  4. Replicability

One of the most important features of memes is how easily spread and replicated they are. While we opted against arguing that being widespread and replicated is a necessary condition for a meme, we do believe that memes need to have the inherent potential for dissemination and replication. Memes are easily replicable because of their form: Individual memes use *meme templates* which can be used and reused for countless different versions of memes. Meme templates are the steady background of the meme, which always remains the same,^10^ while the individual memes can be completed by adding labels or commentary. Often, meme templates are self-explanatory but knowledge of how a meme template functions is part of the necessary background knowledge required to understand a meme. Crucially, it is not only memes themselves which are shared on social media: whole meme templates are shared online as well, aiding the replicability of memes.

Regarding distribution, the fact that memes are mainly spread through social media helps as well. The design of social media platforms makes it likely that memes can easily be spread to a large audience. The fact that memes are easily replicable and widely distributed helps them fulfill an important role within political discourse as well, since it allows for the political message spread by memes to be amplified. The effectiveness of memes at conveying political messaging should not be measured exclusively by how effective individual memes are, but rather, how effective a whole host of memes pushing the same message can be. Because of the ease with which memes can be replicated, communicating through large numbers of memes is easier compared to other speech acts (i.e. it would be harder to outright assert the same message multiple times than circulating memes). Using large quantities of information can make messaging more effective, aided by psychological phenomena such as the repetition effect (Fazio 2020) or impacts to epistemic environments such as epistemic flooding (Anderau 2023).

5. Context Collapse

**Fig. 6:**
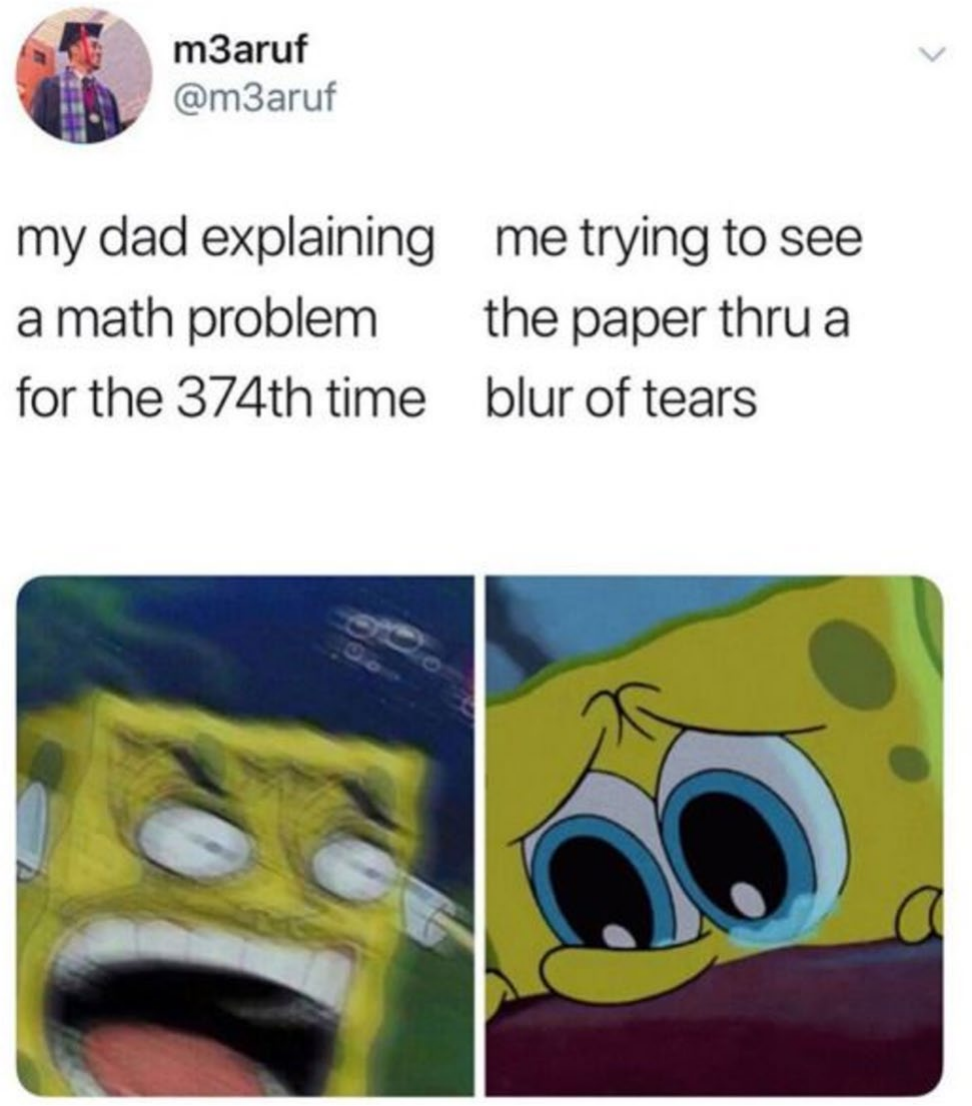
Context collapse in a SpongeBob meme

We identify two levels of context collapse on memes: one operating on the meme itself, and a second one affecting its reception. In communication studies, it has already been described how many memes will often engage in this kind of re-contextualizing of content (Kirner-Ludwig 2022). This fits well with Scott’s (2021) notion of memes as multimodal metaphors: memes will paradigmatically work by collapsing two different, originally unrelated elements. While Scott (2021) analyzes how this is often done through labeling (assigning labels like “me”, “my partner”, etc. to different elements in the picture; see Fig. 1 above), this can also be done through brief descriptions of the context where the image needs to be imagined (*when*-memes, Piata 2022), or brief dialogues that prepare the grounds for a reaction. See, for example, the myriad of memes that pick frames and quotations from SpongeBob and deploy them in a new context (see Fig. 6 for an example). The surprising effect and creativity of the connection, plus the frequent exaggeration, seem to be at the core of the comedic effect.

We take this to be one of the paradigmatic features of memes. Additionally, we identify that memes can also be produced by a second level of context collapse, in which a meme found in a first community is shared in a new one. In this case, the game of context is closer to Marwick and boyd’s (2011) notion of context collapse,^11^ in which different audiences are collapsed into one. Because the audience’s background knowledge grounds the meaning of the meme and its plausible interpretations, altering those same background assumptions will affect the interpretation of the meme. This works in the meme reception more than in its content. While describing the potential positives of meme usage, we will give some more detail on how this works in a particular case.

3. Conditions of reception
  6. Hermeneutical Resource

A hermeneutical resource can be understood, broadly speaking, as a good that allows one to understand one’s own experience: in her classical text, Fricker (2007, p. 158) argues that a lacuna in the “collective hermeneutical resource”^12^ can leave the experience of a disadvantaged person obscure. A paradigmatic hermeneutical resource in Fricker (2007, p. 159) is the concept of “sexual harassment”, but it is not specified that hermeneutical resources restrict themselves to concepts or words. For our purposes, let us pose the question like this: what does it mean for a meme to function as a hermeneutical resource? Under a broad understanding, it means that it can illuminate one aspect of our experience that may have been obscure(d).

In many cases, memes are structured precisely to relate them to a lived experience, or to be “mapping tools for everyday life” (Zidani 2021). This is present in the aforementioned when-memes (Piata 2022), or similar structures like “literally me”. Memes’ capacity to interpret and share one’s own experiences in creative and comic ways is arguably one of the reasons for their popularity. While many of the experiences illustrated in this way by memes tend to be trivial (Fig. 3 above can serve as an example), this opens the way for other memes to illustrate deep reflections.^13^ In the next section, we present a good case that helps illustrate this function (Fig. 9).

7. Low Reputational Cost

Finally, one of the main benefits (from the perspective of the creator and/or sharer of memes) of using memes in political discourse is the fact that memes come at a low reputational cost. As Lynch (2022) argues, it is hard to apply sincerity conditions to memes. Scott (2021) argues that the implicature of a meme is weaker than it is for assertions. Clearly memes allow for a level of plausible deniability which straightforward assertions do not. The similarity of memes to jokes is helpful in this aspect as well. Excuses along the lines of ‘it’s just a joke’/’I was just joking’ translate to memes too: ‘It was just a meme’ seems like a plausible line of defense for someone who is being called out for the content of a meme they have just posted (Lynch 2022).

Therefore memes require less accountability than alternative speech acts which are used in political discourse, especially when compared to assertions. The fact that memes come with a baked-in excuse (‘it’s just a meme’) make them a low-risk communicative strategy within political discussions. Coupled with the many advantages memes have outlined above, it is fair to say that memes are a *low-risk, high-reward* communicative strategy for political discourse. There are benefits to using memes which cannot fully be replicated through assertions or other speech acts but posting a meme at the same time comes at a lower price since there is always a level of plausible deniability.

8. Signaling Function

Because of their humoristic nature, memes frequently exaggerate what they show and draw caricature-like pictures of the world. A question arises: how do sincerity conditions apply to memes? Lynch (2022) is entirely right to center this question in his analysis. He argues that sincerity is not so much about whether the depiction is accurate (maybe it is not supposed to be “accurate”, in the first place), but about the *commitment* that is expressed by sharing it.

This leads us to point out another important function of memes in political discourse.^14^ More than representing the world in a certain way, sharing a meme works to signal someone’s sympathy for a political sensitivity. And *producing* memes, especially in a consistent way, is an even more indelible signal of one’s political sympathy. This is similar to current work by Funkhouser (2017, 2022, 2023), according to which beliefs do not only help us navigate the world, but also function as signals to manipulate others.

On a related note, this function of memes, along with the related features of plausible deniability and humor, make memes great candidates to function as dog whistles. A dog whistle is “speech that seems ordinary but sends a hidden, often derogatory message to a subset of the audience” (Quaranto 2022).

In the context of memes, the best example is Pepe the Frog. Originally intended as a non-political character, Pepe the Frog has over the years been co-opted by ‘alt-right’ sympathizers (Glitsos and Hall 2020). Pepe the Frog has been famously used to represent Donald Trump, other so-called ‘alt-right’ politicians, and even Nazi characters. This reflects the aforementioned point of the historicity of functions: while the character of Pepe the Frog was not designed to function as an alt-right (or far right) dog whistle, it has acquired this property over time by evolution.

**Fig. 7:**
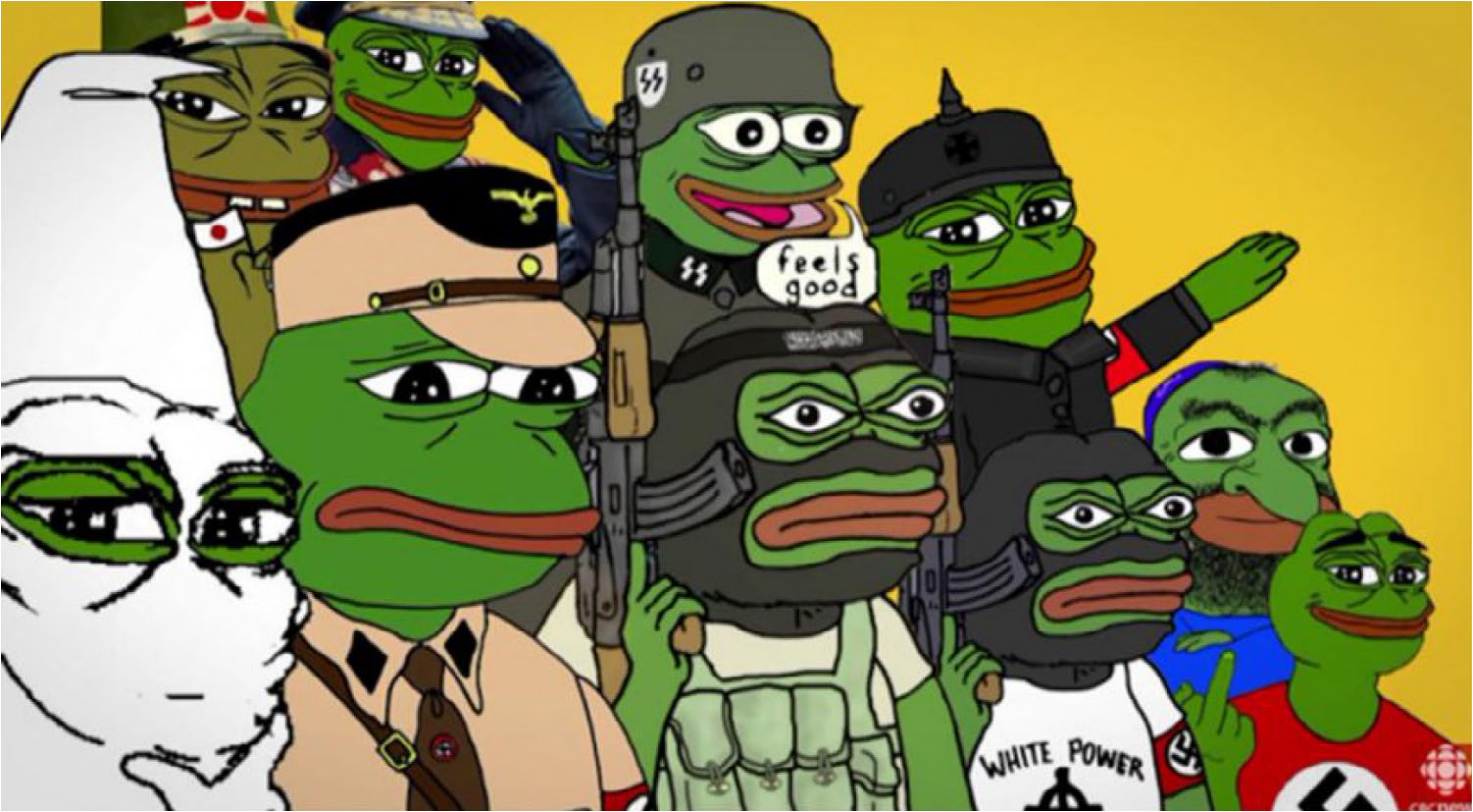
Pepe the frog incarnating several far-right agents. Meme retrieved from a tweet (It can be found in: https://twitter.com/hip_thrust_fan/status/1779558288381587695) celebrating the presentation of a far-right movement in Spain

**Fig. 8:**
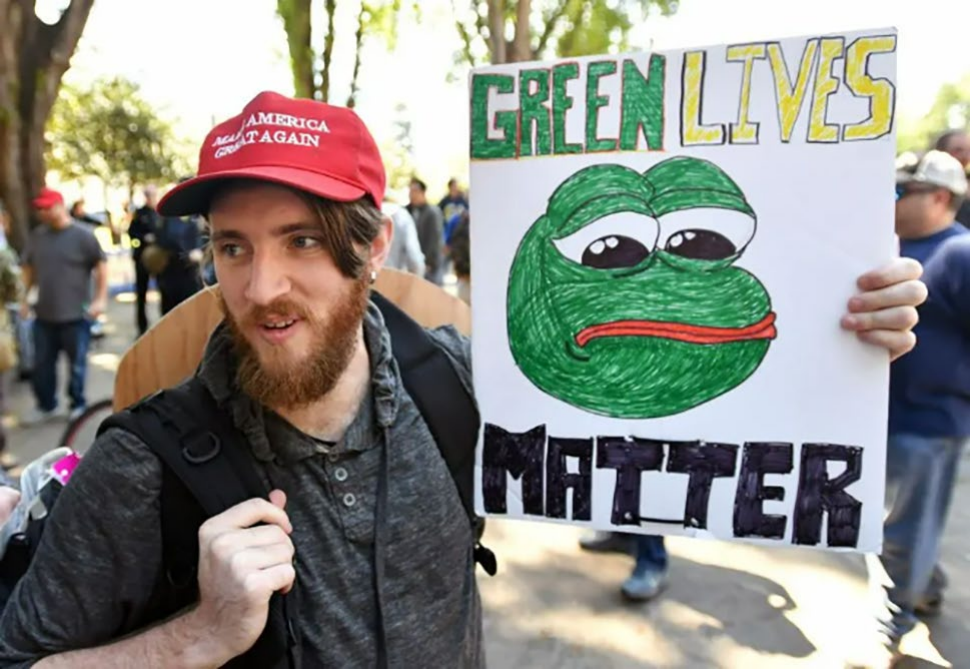
Pepe the Frog signaling alt-right affiliations, similar to the ‘Make America Great Again’ slogan next to it. While mocking the Black Lives Matter movement, this instance avoids becoming overt hate speech. Original photography from Josh Edelson. Found in Placido (2017)

While we acknowledge potential controversies for displaying hate symbols, we have chosen to include Fig. 7 (see below) to show the extremes to which Pepe the Frog can signal far right sympathies. Precisely because of the meme’s function as a dog whistle, it’s important to have a clear picture of the positions that it can signal. However, more subtle instances like Fig. 8 are better suited to function as dog whistles without conveying a straightforward hate message.

## The Good, The Bad and the Ugly

We’ve established that memes are *low-risk, high-reward* communicative acts, especially within political discourse. This makes them effective for political messaging and can explain their increased popularity for the dissemination of political messaging. We do not want to argue that this is inherently good or bad: Depending on how memes are used they could be used for noble purposes (both from an epistemic and a moral perspective) or exploited by more nefarious actors. What we do believe is that both for the positive and negative uses of memes in political discourse, their impact will be high: Because of the reasons outlined above, memes are extremely effective regardless of whether they are used in a positive or negative sense. In this section, we try to outline the good, the bad and the ugly when it comes to the use of memes for political messaging.

### The Good

We have mentioned that memes function as tools to make sense of our own experiences and communicate them. While we have pointed out that this can be the case for all kinds of users, now we want to show how this can be especially convenient for communities who live in hermeneutical marginalization.

Take the case^15^ analyzed by Vizuete and Barbarrusa (forthcoming). Briefly put, they contend that patients with Cystic Fibrosis typically live with severe symptoms that, among other complications, involve an important reduction of their life expectancy. In the U.S., the median predicted survival age for patients born in the 1990s is 31 years old (Medical News Today 2019, Life expectancies by birth year section). From an epistemic point of view, this implies that common hermeneutical resources, like “being young”, “healthy”, or “mid-life crisis” don’t work in the same way they do for the general population. These are important concepts to orient oneself through different stages of life, and living without them conveys epistemic and practical difficulties. In their paper, they study how certain isolation conditions in an online community meant to share memes related to this condition help in recognizing and repairing these hermeneutical gaps.

**Fig. 9:**
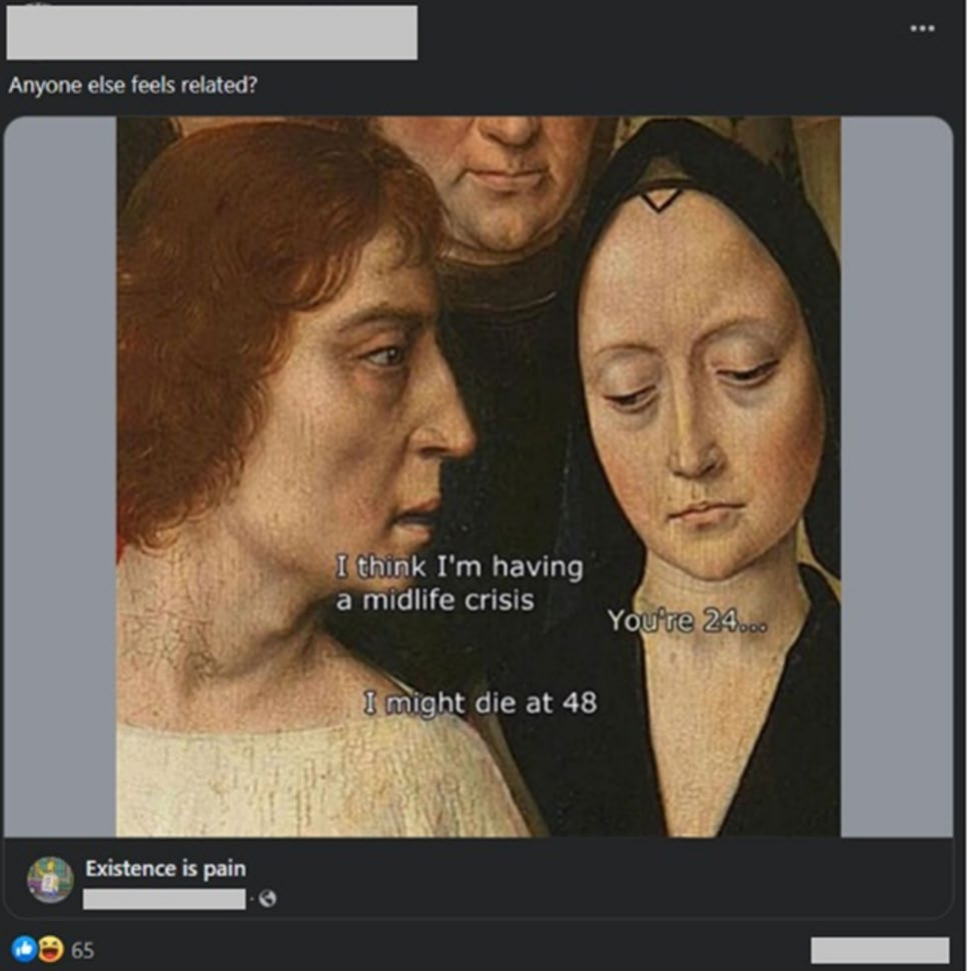
‘Midlife Crisis’ Meme, as shown by Vizuete and Barbarrusa (forthcoming)

They provide the following example (Fig. 9):

While we refer to that paper for a more detailed discussion of the case, we see several of our functions put to good purposes here. Namely, they address the function of context collapse in the following way:

Here we don’t find any explicit reference to Cystic Fibrosis. Instead, the meme is originally found on the ‘Existence is pain’ community.

In this case, the assumed background knowledge makes clear that this is a special context, different from the one in which the original meme was found. The meme found on ‘Existence is pain’ seemingly exploits context collapse (boyd, 2008; Frost-Arnold 2021) for humor, perhaps conflating the low life expectancy in the Middle Ages with an accelerated pace of life in current times. Nevertheless, when the meme is shared in the CF-related group, it is reappropriated through a second level of context collapse. Leveraging Grice’s (1975) maxim of relevance, this invites us to reconstruct the meaning of the meme to make it relevant to the group’s discussion. ‘Middle-life crisis’ or expecting to live until 48 are no longer about the Middle Ages or generic worries, but very tangible realities for community members.

This kind of context collapse, we add, helps in making visible the different interpretations between insiders (CF patients) and general audiences outside the group. What is comedy through exaggeration for the latter, is comedy through painful truth for the former.

The authors also describe how the rules of isolation in this particular community are at the service of dark humor, enabling the first function we have shown. Indeed, the humor in this group exploits much of the insiders’ background knowledge on their shared experiences with the illness: symptoms and hermeneutical gaps that are hardly experienced in a similar way by others. Apart from fostering belonging to the community, humor favors establishing new connections that will help in the production of these hermeneutical resources.

### The Bad

The use of memes in political discourse can also have negative consequences. In this section (*The Bad*) we will consider negative consequences which can arise as an unintentional side effect of meme usage, whereas the next section will explore how memes can be deliberately exploited for nefarious purposes (*The Ugly*). Even if people post memes without harmful intentions, the proliferation of memes in political discourse can have adverse effects. Two worries seem particularly poignant in this regard: (1) The negative effects of an in-group/out-group dynamic and (2) the removal of nuance from political debates.

Regarding the first point, it seems almost certain that creating a strong in-group identity leads to the exclusion of the ‘out-group’. How harmful this is will of course depend on the context: It does not seem inherently worrisome if a marginalized group (e.g. the cystic fibrosis community discussed above) creates an in-group dynamic at the cost of excluding mainstream society as the outgroup. This might even be true if the out-group dynamic is actively created as well, for instance by negatively remarking the lack of understanding for the disease outside of the community. However, even in these cases there is a danger that in-groups can become entrenched in echo chambers or at least epistemic bubbles (Nguyen 2020). This worry becomes even more pressing if the in-group does not consist of a marginalized community.

If the in-group is a more dominantly situated social group and the out-group is a marginalized community this dynamic becomes troublesome even if there is no malice behind the memes themselves. Strengthening an in-group/out-group dynamic which runs along the lines of dominant and marginalized power structures is inherently worrisome. Of course, this dynamic can be deliberately exploited. But it is reasonable to assume that most people who post memes do so without intending any harm.

The second harmful effect is the removal of nuance from political debates once this discourse relies heavily on the use of memes. As discussed earlier, memes are by default not an ideal medium for nuanced discussion. In order to effectively communicate a political message through the meme format, it is necessary to remove a lot of context from the debate. This context ‘ellipsis’ is often filled with the background knowledge and assumptions which the consumer of a meme needs to have in order to understand the meme.

Given the caricature function memes play in political discourse, the points which memes are designed to make are short and simple. Again, even the caricature function of memes does not need to be used with bad intentions: To a certain extent, memes can offer meaningful and poignant political commentary even as caricatures. The problem arises when memes become ubiquitous in political debates and make up a large percentage of the discourse. An individual meme will not be harmful in this context, but a barrage of memes or a debate in which both sides communicate heavily through memes is. ‘Meme wars’ (Al-Rawi 2021; Bogerts and Fielitz 2018; Denisova 2019; Hannan 2018) are not an effective tool for nuanced political debate, but they do lend themself to political point scoring and riling up one’s own in-group. Even if full-blown meme wars can be avoided, we should be worried not by the use of individual memes in political debate, but once memes make up a significant part of the discussion, which they arguably already do (Al-Rawi 2021; Bogerts and Fielitz 2018; Denisova 2019; Hannan 2018; Lynch 2022). This is true even if the memes are posted without nefarious intent and without targeting marginalized communities.

### The Ugly

The effectiveness of memes as a tool for political discourse can of course also be harnessed deliberately by nefarious actors. This is particularly true for the in-group/out-group dynamic which memes are effective at creating and promoting. As mentioned above, we are especially concerned if the out-group being deliberately ostracized is one which is already marginalized or discriminated against in society at large. Memes can easily be made at the expense of marginalized communities and purposefully used to other them.

Because of their humoristic nature, memes can also be used to make extreme content more palatable to a mainstream audience which might reject it if it was asserted more straightforwardly. The characteristics we mentioned above which make memes effective tools for political messaging all become worrying in the hands of unscrupulous actors whose goal is to harm their political opponents, especially if these are made up of marginalized communities.

The fact that posting a meme is possible to almost any social media user and that there is a low reputational cost attached to the act of posting a meme means that these negative effects can easily become more pronounced. Even for nefarious political actors, the hurdle to posting a meme is very low, it is a low-effort communicative act. At the same time, it is also a speech act which has a high likelihood of achieving strong results in political debates. It should be noted that some of the negative effects which could be unintentional side effects of meme usage, such as driving polarization or removing nuance from political debates can also be deliberately sought out by political actors with ill intentions. Even if memes are not effective at directly convincing others, they could be used intentionally to further division and undermine good faith political discourse.

In the following section, we will examine how memes can be a driving factor in turning political debates toxic and how this characteristic can deliberately be exploited. We use the Gamergate movement and the manner in which the 2016 Trump presidential campaign co-opted it as a case study to illustrate this point. The Gamergate movement started as backlash to a supposed ethics scandal within gaming journalism. Despite the initial infractions being relatively minor, the hashtag ‘#gamergate’ became viral and led to larger discussions of and pushback against feminism and the role of women within the industry, including the targeting, doxing and harassment of women at the heart of the initial story, such as Anita Sarkeesian, Zoë Quinn, and Brianna Wu (Romano 2021).

**Fig. 10:**
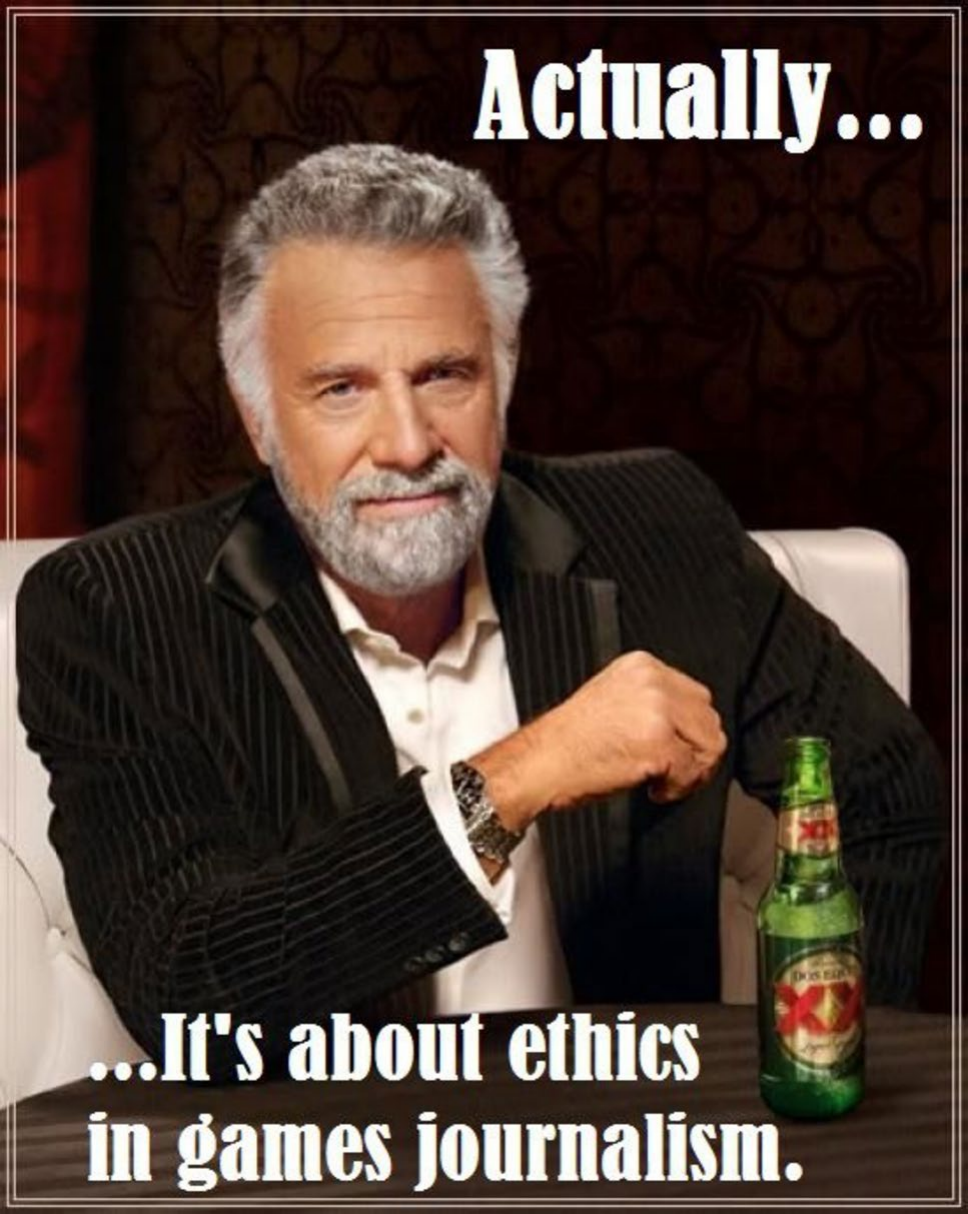
Is a simple meme supporting the initial Gamergate movement, which backs the notion that the driving force behind the movement was merely about addressing ethics violations in gaming journalism

**Fig. 11:**
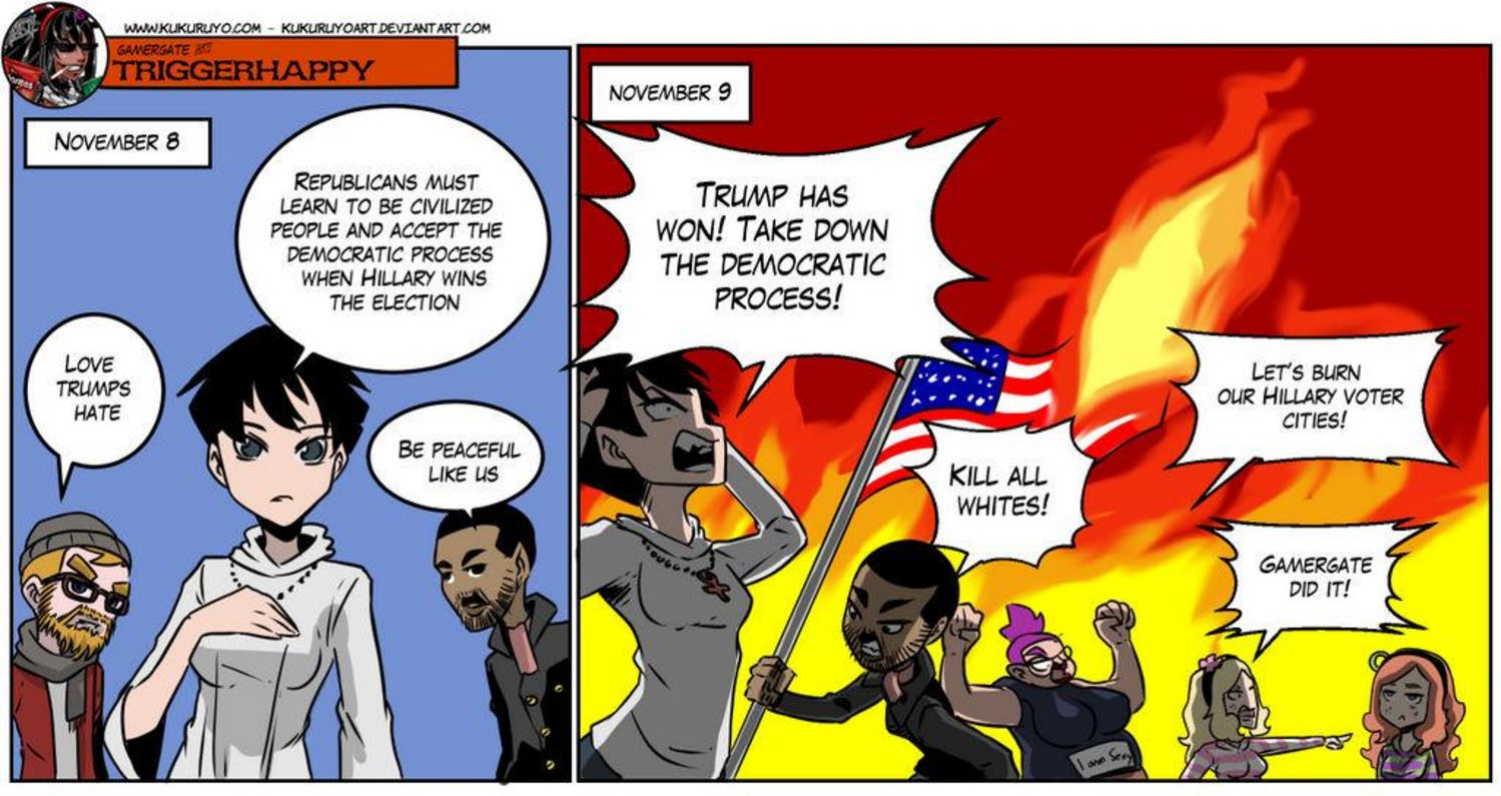
Is a meme which shows how the target audience for the Gamergate movement and the 2016 Trump presidential campaign showed significant overlaps. Although Gamergate is more peripheral to this meme, it is explicitly mentioned as a reference in this political meme

Interestingly enough, the movement was deliberately courted by the Trump 2016 presidential campaign according to its chief advisor Steve Bannon (Snider 2017; Romano 2021). In general, memes are used on platforms such as 4 chan as a deliberate strategy by the alt-right to make fringe political positions such as white supremacism more palatable to mainstream audiences (Arthur 2020). The book *It Came from Something Awful* by Dale Beran (2019) offers a good historical overview of this development. Figures 10 and 11 below offer an illustration of this process.

## Outlook and Possible Solutions

We have established that using memes is a low-risk, high-reward communicative strategy in political discourse. Their impact is strong regardless of whether they are used positively or negatively. The question therefore is what should be done in order to preserve the positive effects of meme usage while negating its detrimental force. In particular, we should also be worried about the role memes play in the spread of misinformation (Lynch 2022). However, since memes are not assertions or testimony in the classical sense, we need to see in what sense memes are comparable to misinformation in the first place. We do share Lynch’s (2022) worries about memes contributing to harmful political content, but want to offer an explanation of both the political as well as the epistemic impact memes can have in this regard. The question will of course require a multi-layered response: There is no magic bullet which will prevent the negative impact memes can have in political discourse.^16^

The one solution we want to elaborate upon here is the following: We need to adapt the norms governing memes as a speech act. Since memes are a relatively novel form of communication, it stands to reason that our norms regarding memes are not yet set in stone and remain unclear. We have posited that memes come at a presumably low reputational cost, especially when compared to assertions. Given the momentous impact memes can have on political discourse, it seems questionable why they should come at such a low reputational cost. Our suggestion therefore is the following:

*Memes should be seen as powerful speech acts with the potential to convey strong political messages. While memes are very different from assertions, the commitment and reputational cost attached to them should at the very least be equally weighty.*

This proposal requires several clarifications and explanations:

### What do we mean by memes as a ‘speech act’? Is it creating a meme, posting a meme, sharing or liking a meme or even creating the meme template itself?

It is important to distinguish between the different potential acts which are made possible by memes. Creating a meme template is not the same as creating an individual meme. While there are normative issues which apply to creating a meme template, creating the template is usually a much more neutral act. The norms which regulate this action would mainly be moral in nature, i.e. there might be concerns about turning images of people into a meme without their knowledge or even against their will, especially if the subject of the meme is a child. Certain meme templates can already hint at their content and contribute to polarization or degrade political discourse regardless of the captions added to it.

Nevertheless, such instances are rare. We therefore want to focus mainly on the acts of creating and especially of posting a meme. The emphasis on posting memes is justified by the fact that this is by far the most common speech act involving memes. Creating a template is much rarer but even creating an individual meme requires significantly more effort than sharing a meme. Because memes are easily replicated and spread, most people who post memes are not necessarily the creators of the meme itself. As such, posting a meme seems to be the primary communicative act involving memes and the one which is most interesting for us. We can assume that in terms of reputational cost, whatever holds for posting a meme would also apply for creating one since the creator has less plausible deniability. The creator of the meme template however, should in normal circumstances not be made liable for all the memes the template generates.

One interesting question is whether sharing or liking a meme holds the same weight as posting a meme. On the one hand, especially for sharing, the actions seem very closely comparable provided one is not the creator of the meme as well. On the other hand, there is some controversy if we really are equally responsible for content we share in novel speech acts online in the case of retweeting (Arielli 2018; Marsili 2021). Nevertheless, we are inclined to say that the norms governing sharing and liking memes are similar to those of posting memes. Therefore, we think it is best to focus on the norms of posting a meme, while assuming that similar norms would apply to creating (at least from a minimal perspective), sharing and liking a meme. If a reader is inclined to disagree, they can read this analysis as strictly applying to the act of posting a meme.

*Even if we wanted to, how could we hold people accountable for posting a meme? Posting a meme is not the same as asserting something so it is unclear how we can hold people accountable for it and attach a reputational cost to this act as we would for assertions?*

We concur that posting a meme is not the same as asserting its content. Memes cannot be treated the same way assertions are and the implicature of a meme is at the very least diluted because memes are subject to different interpretations (Scott 2021). This is why traditional methods of combating fake news or misinformation, such as fact-checking would not be effective with regards to memes.^17^ Lynch (2022) argues that there is a different way in which we can hold meme posters accountable: While posting a meme does not necessarily pose an epistemic commitment to the content, we can still read them as a *political commitment*. And we agree with Lynch (2022) that memes do have a relatively clear message, one which is meant to be understood clearly, at least by the intended target audience. While posting a meme might be less clear than an outright assertion, memes have clearly intended take-away messages.

Nevertheless, we maintain that memes have an important part to play *epistemically* as well. If memes had no impact on the epistemic standing of their audience and could not be used to convince others of political arguments, they would not be such a popular and effective tool for political discourse. The question is how we can account for the epistemic effects of memes while maintaining that memes do not function the same way as assertions. It seems strange to say that people derive knowledge or even beliefs from memes the same way they do from regular testimonial sources. Deferring to testimony is a viable source of second-hand knowledge, but it seems strange to defer to a meme in this way: ‘I know p because a meme told me so’ seems like a very unnatural stance to take.

The solution we propose is to say that memes can impact the epistemic standing of its audience through *propagation* rather than the transmission found in regular testimony cases. We use Alisson Hills’ (2020) account of *propagation*, which she originally developed in order to describe how we learn from moral testimony as opposed to regular testimony. In her account, Hills distinguishes between transmission, which can lead to second-hand knowledge through regular testimony, and propagation, which can lead to first-hand knowledge and understanding through moral testimony. We believe that the propagation model outlined in Hills (2020) can be fruitful to explain the epistemic impact of memes as well. While transmission allows for the most straightforward transfer of knowledge through testimonial sources, Hills (2020) highlights that there are several advantages to propagation. One is that propagation can lead to the creation of first-hand knowledge, whereas transmission will only induce second-hand knowledge. Propagation is not the *grounds* for knowledge, but it can motivate inquiry, which can then lead to first-hand knowledge.

We believe that a similar thing applies to memes: It seems strange to say that we know something because of a meme—as would be the case in the transmission model—, but seeing a meme could still prompt us to inquire and reflect more deeply on a question, even if we are not able or willing to acknowledge that the meme prompted our inquiry. Importantly, in the cases Hills (2020) describes, the moral testimony usually consists of metaphors or analogies. This is another similarity with memes, which are interpreted as metaphors as well (Scott 2021).

Another advantage of propagation is that it can further epistemic goals other than merely gaining knowledge: Propagation is able to lead to understanding, which can be more valuable than second-hand knowledge depending on our situation (Hills 2020). Importantly, understanding cannot simply be transmitted through testimony. If we consider memes as a tool within political discourse, it is an important addition to testimony and straightforward assertions, since inducing first-hand knowledge and understanding can be more advantageous in some cases.

Importantly, even though Hills (2020) outlines the advantages of propagation in terms of effectiveness, it is important to keep in mind that this effectiveness is neutral with regards to the outcome: It can be used both for beneficial and harmful purposes. Propagation can also be effective at spreading harmful viewpoints, which is another similarity to memes: They can both use propagation to induce first-hand knowledge, beliefs and understanding in cases in which the outcome is morally or epistemically harmful. Another similarity between moral testimony and memes is that they can trigger an emotional response in a way in which transmission cannot. This is again possible through propagation. An emotional response could of course also trigger epistemic consequences downstream. If memes lead us to form a sub-doxastic state, such as an alief (Gendler 2008), this might influence and bias us in future inquiries and impact our belief formation process.

Therefore, memes can become extremely effective tools for political discourse through propagation. This explains why we worry about the contribution of memes to misinformation and disinformation, even though memes are not assertions or classical examples of testimony (Lynch 2022). While we believe that it is necessary to carefully distinguish between memes and misinformation for this reason, memes can help promote the same messages as misinformation. And through propagation, they can be more effective at spreading such contents than fake news and misinformation, because it can prompt inquiry. While less direct than transmission, this can lead to a wider range of epistemic outcomes, including first-hand knowledge and understanding.

Arguably, memes have contributed more heavily to political phenomena such as vaccine skepticism during the Covid-19 pandemic than fake news, because memes were much more widespread and reached a larger audience than fake news (Altay et al. 2023). And our framework explains why Lynch (2022) would be worried about the contribution of memes to misinformation, while also disentangling memes from misinformation proper by explaining how it can have an epistemic impact through propagation rather than transmission.

### Do Memes Really Come at a Low Reputational Cost?

One open question is whether memes really come at a low reputational cost for the person posting a meme. While we believe that this is an intuitively appealing proposition, it could of course be the case that the real-life norms governing memes are much stricter than we have assumed. In this case our proposed sharpening of the norms of posting memes would become redundant, not because the proposed norms are wrong, but because these norms are in fact already in place. While we are confident in our claim that memes come with a level of plausible deniability which ordinary assertions do not possess, this is ultimately an empirical claim which we cannot prove.

Unfortunately, despite there being a vast literature on memes outside of philosophy, there is no data to be found on this specific question. While finding such data lies beyond the scope of this theoretical paper, we do believe this would be a fruitful avenue for future research, both for other empirical academic disciplines or for experimental philosophers within philosophy itself.

In general, it would be great to have more data on the impact and reception of memes. There is also a dearth of large scale empirical data on the use of memes in general as well as in political contexts. Empirical research usually focusses on the use of memes in highly specific niche contexts but does not provide clear answers on how often memes are used in political discourse. Since we believe that the impact of memes becomes more pronounced when we are confronted with a whole host of memes, rather than just individual instances of them, it would be especially interesting to study the difference between the reception of singular memes compared to groups of memes. We hope that, in addition to the theoretical and normative points we have argued for, our paper can function as a basis for future empirical research on these questions.

## Conclusion

Despite the growing popularity of memes in the digital landscape, the philosophical work on Internet memes remains scarce. We hope to fill this gap by acknowledging previous contributions, including the more extensive literature on memes outside of philosophy and adapting them to a definition of Internet memes. Using real examples of memes, we’ve proposed a functionalist account of memes: an Internet meme is a piece of digital content that is particularly apt to fulfil eight different functions that we identify. We then move on to explain how these functions of memes can affect political discourse in different ways—for good, for bad, or for worse. Our account can explain why memes have become increasingly important in political conversations around the world. Importantly, our paper takes a neutral view on the role of memes—highlighting both positive and negative impacts memes can have in this context. In the final part of the paper, we have also presented an avenue for future research on (the political impact of) memes, both within and outside philosophy.

## Footnotes

1 Dawkins has occasionally addressed the topic of internet memes (Fazal 2018), (Solon 2013). Nevertheless, in these cases he has underscored how they fit the general features of his own broader notion of memes, without analyzing their peculiarities of humor or conditions of production. In her public outreach, Blackmore (2011) has addressed internet memes as well, including their humoristic nature and video variants. Our paper, nevertheless, hopefully contributes to bringing these insights to academic philosophy.

2 For a philosophical discussion of memes in Dawkins’ sense, see Dennet (1990).

3 The special issue in *Information, Communication & Society* (Mortensen and Neumayer 2021) provides a great discussion on the ‘playful politics of memes’. See Xie (2022) for a discussion on the pragmatics of internet memes.

4 For a more in-depth discussion of the politics of online discourse, see Frost-Arnold (2023).

5 For readers interested in a more straightforward definition of memes, Scott (2021) is recommended. For definitions outside of philosophy, (see Amir 2021; Shifman 2013; Rogers and Giorgi 2024; Castaño 2013).

6 Fallis refers to Krohs and Kroes (2009) for more careful debates on the notion of function.

7 We thank Antti Kaupinen for raising this point.

8 Since we do not want to restrict our analysis to object labeling memes as Scott (2021) does, we are open to media other than text and image, such as videos or gifs to be part of the mix of different media.

9 The interrelation between memes and online environments also implies that memes are likely to be accompanied by technological affordances (like reaction buttons and invitations to share them) that will not necessarily be present in jokes.

10 Some meme templates do allow for small variations amongst the template, such as an extended version of a meme allowing for more labels to be added.

11 Context collapse has commonly been seen as an ethical or epistemic danger (Frost-Arnold 2021): the rules governing a speech act in one audience can be misunderstood or ignored in a different one. Consequently, a message that could have an ironic meaning in one environment, can be taken at face value in another, with all the misunderstanding and conflicts this may raise. Nevertheless, we believe that context collapse may bring new possibilities for creativity and perhaps what Medina (2013) calls epistemic friction.

12 See Goetze (2018) for detailed debates on how it should be understood that hermeneutical resources (or ‘interpretive tools’) are shared.

13 This is not to say that every meme that follows a when-structure or “literally me” format will provide a useful interpretation of a lived experience. In fact, as Falbo (2022) shows, some hermeneutical resources (Falbo thinks of oppressive concepts) can easily do the opposite, obscuring more accurate ones.

14 While the signaling function of memes is particularly likely to appear in political contexts, it can occur in apolitical memes as well. Supporters of certain musical artists or fandoms (for example, a videogame or book series) can signal their affinity in this way. Of course, some fandoms are openly political (e.g.: punk rock bands), but others are much more apolitical (e.g.: being a *Star Wars* fan).

15 Their paper includes a section to explain their efforts to study the case following Frost-Arnold’s (2023) guidelines for ethical research of online disadvantaged communities.

16 Discussing all the possible solutions would extend beyond the scope of this paper, but we will briefly mention two in this footnote: (1) Holding the corporations which run social media platforms to task in order to provide better content moderation, including for memes. We defer to Frost-Arnold (2023) for a detailed discussion on how to improve content moderation on social media (2) Bettering our epistemic environments online to mitigate the negative effects of meme usage. Ryan’s (2018) notion of *epistemic environmentalism* is a useful framework in this regard.

17 There are of course also arguments that fact-checking is not even a particularly effective tool against fake news or misinformation (Jaster and Lanius 2019). While we believe these arguments are convincing, fact-checking is still regularly employed against misinformation but it seems fair to point out that using fact-checking to combat memes is a non-starter.

## References

[CR1] Al-Rawi A (2021) Political memes and fake news discourses on instagram. Media Commun 9(1):276–290. 10.17645/mac.v9i1.3533

[CR2] Altay S, Berriche M, Acerbi A (2023) Misinformation on misinformation: conceptual and methodological challenges. Social Media + Society 9(1). 10.1177/20563051221150412

[CR3] Amir M (2021) What are internet memes and how they are used for different purposes? Available at SSRN: https://ssrn.com/abstract=3874090 or 10.2139/ssrn.3874090

[CR200] Anderau G (2023) Fake news and epistemic flooding. Synthese 202(4):106. 10.5167/uzh-237567

[CR4] Arielli E (2018) Sharing as speech act. Versus. 127, 2/2018, 243–258.

[CR5] Arthur R (2020) The man who helped Turn 4Chan Into the Internet’s Racist engine. Vice. https://www.vice.com/en/article/m7aap8/the-man-who-helped-turn-4chan-into-the-internets-racist-engine

[CR6] Beran D (2019) It came from something awful: How a toxic troll army accidentally memed Donald Trump into office. All Points Books.

[CR7] Blackmore S (2000) The meme machine. Oxford University Press, Oxford

[CR8] Blackmore S (2011) A great internet meme's never gonna give you up. The Guardian. https://www.theguardian.com/commentisfree/2011/mar/18/internet-meme-never-gonna-give-you-up

[CR9] Bogerts L, Fielitz M (2018) “Do you want meme war?” Understanding the visual memes of the German far right. In: Maik F, Nick T (eds) Post-digital cultures of the far right: Online actions and offline consequences in Europe and the US.137–153. Majuskel Medienproduktion, Berlin

[CR10] boyd D (2008) Taken out of context: American teen sociality in networked publics. PhD Thesis, University of California, Berkeley, CA.

[CR11] Castaño DCM (2013) Defining and characterizing the concept of Internet Meme. Revista CES Psicología 6(2):82–104

[CR12] Cohen T (1999) Jokes: Philosophical thoughts on joking matters. University of Chicago Press, Chicago

[CR13] DaFuq!?Boom!. (2023) Skibidi Toilet - Season 1 [FULL SCREEN] [Video]. YouTube. https://www.youtube.com/watch?v=WePNs-G7puA

[CR14] Dawkins R (1976) The selfish gene. Oxford University Press, Oxford

[CR15] Denisova A (2019) Internet memes and society: social, cultural, and political contexts. Routledge, London

[CR16] Dennett DC (1990) Memes and the exploitation of imagination. J Aesthetics Art Criticism 48(2):127–135

[CR17] Diedrichsen E (2022) On the interaction of core and emergent common ground in Internet memes. In: Xie C (ed) The Pragmatics of Internet Memes. John Benjamins Publishing Company, Amsterdam, pp 85–121

[CR18] Falbo A (2022) Hermeneutical injustice: distortion and conceptual aptness. Hypatia 37(2):343–363

[CR19] Fallis D (2015) What is disinformation? Libr Trends 63(3):401–426

[CR20] Fazal M (2018) Talking to the guy who invented the word 'Meme': Richard Dawkins. Vice. https://www.vice.com/en/article/d35ana/talking-to-the-guy-who-invented-the-word-meme-richard-dawkins

[CR21] Fazio LK (2020) Repetition increases perceived truth even for known falsehoods. Collabra Psychol 6(1):38. 10.1525/collabra.347

[CR22] Fricker M (2007) Epistemic injustice: power and the ethics of knowing. Oxford University Press, Oxford

[CR24] Frost-Arnold K (2021) The epistemic dangers of context collapse online. In: Lackey J (ed) Applied epistemology. Oxford Academic, Oxford.

[CR23] Frost-Arnold K (2023) Who Should We Be Online? A Social Epistemology for the Internet. Oxford University Press, New York

[CR25] Funkhouser E (2017) Beliefs as signals: a new function for belief. Philos Psychol 30(6):809–831. 10.1080/09515089.2017.1291929

[CR26] Funkhouser E (2022) A tribal mind: Beliefs that signal group identity or commitment. Mind Lang 37(3):444–464

[CR27] Funkhouser E (2023) Dangerous beliefs, effective signals. Philos Psychol 36(5):969–989. 10.1080/09515089.2022.2101444

[CR28] Gendler TS (2008) Alief in action (and reaction). Mind Lang 23:552–585. 10.1111/j.1468-0017.2008.00352.x

[CR29] Glitsos L, Hall J (2020) The Pepe the Frog meme: an examination of social, political, and cultural implications through the tradition of the Darwinian Absurd. J Cult Res 23(4):381–395. 10.1080/14797585.2019.1713443

[CR30] Godwin M (1994) Meme, counter-meme. Wired. https://www.wired.com/1994/10/godwin-if-2/

[CR31] Goetze TS (2018) Hermeneutical dissent and the species of hermeneutical injustice. Hypatia 33(1):73–90

[CR32] Graham P (2010) Testimonial entitlement and the function of comprehension. In: Haddock A, Miller A, Pritchard D (eds) Social epistemology. Oxford University Press, New York, pp 148–174

[CR33] Grice HP (1975) Logic and conversation. In: Maite E., Stainton RJ (eds) The semantics-pragmatics boundary in philosophy. Broadview Press, Canada, pp. 47.

[CR34] Hannan J (2018) Trolling ourselves to death? Social media and post-truth politics. Eur J Commun 33(2):214–226

[CR35] Heritage A (2000) The American Heritage dictionary of the English language, 4th edn. Houghton Mifflin, Boston

[CR36] Hills A (2020) Moral testimony: transmission versus propagation. Philos Phenomenol Res 101:399–414. 10.1111/phpr.12595

[CR37] Jaster R, Lanius D (2019) Die Wahrheit Schafft Sich Ab: Wie Fake News Politik Machen. Ditzingen: Reclam.

[CR38] Kirner-Ludwig M (2022) Internet memes as multilayered re-contextualization vehicles in lay-political online discourse. In: Xie C (ed) The pragmatics of internet memes. John Benjamins Publishing Company, Amsterdam, pp 145–181

[CR39] Krohs U, Kroes P (eds) (2009) Functions in biological and artificial worlds. MIT Press, Cambridge, MA

[CR40] Lynch MP (2022) Memes, misinformation, and political meaning. South J Philos 60(1):38–56. 10.1111/sjp.12456

[CR41] Marsili N (2021) Retweeting: its linguistic and epistemic value. Synthese 198:10457–10483

[CR42] Marwick AE, boyd D (2011) I tweet honestly, I tweet passionately: twitter users, context collapse, and the imagined audience. New Media Soc 13(1):114–133. 10.1177/1461444810365313

[CR43] Medical News Today (2019) Cystic fibrosis life expectancy: Averages by stage and age. https://www.medicalnewstoday.com/articles/326316?c=849353037480#life-expectancies-by-birth-year

[CR44] Medina J (2013) The epistemology of resistance: gender and racial oppression, epistemic injustice, and resistant imaginations. Oxford University Press, Oxford

[CR45] Mortensen M, Neumayer C (2021) The playful politics of memes. Inf Commun Soc 24(16):2367–2377. 10.1080/1369118X.2021.1979622

[CR46] Nguyen CT (2020) Echo chambers and epistemic bubbles. Episteme 17(2):141–161. 10.1017/epi.2018.32

[CR47] Nguyen CT (2021) Twitter, the intimacy machine. The Raven (online).

[CR48] Piata A (2022) Stylistic humor across modalities. In: Xie C (ed) The pragmatics of internet memes. John Benjamins Publishing Company, Amsterdam, pp 36–63

[CR49] Placido DD (2017) How “Pepe The Frog” Became A Symbol Of Hatred. Forbes. https://www.forbes.com/sites/danidiplacido/2017/05/09/how-pepe-the-frog-became-a-symbol-of-hatred/

[CR50] Quaranto A (2022) Dog whistles, covertly coded speech, and the practices that enable them. Synthese 200(4):1–34

[CR51] Rogers R, Giorgi G (2024) What is a meme, technically speaking? Inf Commun Soc 27(1):73–91. 10.1080/1369118X.2023.2174790

[CR52] Romano A (2021) What we still haven’t learned from Gamergate. Vox. https://www.vox.com/culture/2020/1/20/20808875/gamergate-lessons-cultural-impact-changes-harassment-laws

[CR53] Ryan S (2018) Epistemic environmentalism. J Philos Res 43:97–112

[CR54] Schmid UK, Schulze H, Drexel A (2024) Memes, humor, and the far right’s strategic mainstreaming. Inform Commun Soc, 1–20. 10.1080/1369118X.2024.2329610

[CR55] Scott K (2021) Memes as multimodal metaphors. Pragmat Cogn 28(2):277–298

[CR56] Shifman L (2013) Memes in a digital world: reconciling with a conceptual troublemaker. J Comp-Mediated Commun 18(3):362–377. 10.1111/jcc4.12013

[CR57] Snider M (2017) Steve Bannon learned to harness troll army from 'World of Warcraft’. USA Today. https://eu.usatoday.com/story/tech/talkingtech/2017/07/18/steve-bannon-learned-harness-troll-army-world-warcraft/489713001/

[CR58] Solon O (2013) Richard Dawkins on the internet's hijacking of the word 'meme'. Wired. https://www.wired.com/story/richard-dawkins-memes/

[CR100] Vizuete LM, Barbarrusa D (forthcoming). Am I Still Young at 20? Online Bubbles for Epistemic Activism. Topoi

[CR59] W K (2009) Demotivational Posters. Know Your Meme. https://knowyourmeme.com/memes/demotivational-posters

[CR60] Xie C (2022) The pragmatics of internet memes. John Benjamins Publishing Company, Amsterdam

[CR61] Zidani S (2021) Messy on the inside: internet memes as mapping tools of everyday life. Inf Commun Soc 24(16):2378–2402. 10.1080/1369118X.2021.1974519

